# Acute respiratory failure following shunt disconnection

**DOI:** 10.1186/1743-8454-7-S1-S52

**Published:** 2010-12-15

**Authors:** Robert Adderley

**Affiliations:** 1 Pediatric Critical Care The University of British Columbia Room 2L5 British Columbia's Children's Hospital 4480 Oak Street Vancouver, British Columbia V6H 3V4, Canada

## Background

A sixteen year old boy with a Meningomyocele/Arnold Chiari Malformation was admitted to the Emergency Department of a large community regional hospital, with acute respiratory failure. His back defect had been closed shortly after birth and a ventriculoperitoneal (VP) shunt inserted. He had had only one shunt revision, when he was thirteen months old, and he underwent an uneventful scoliosis repair at twelve years of age for a significant scoliosis.

## Materials and methods

He became ill with a clinical viral upper respiratory tract infection, which evolved into bilateral pneumonia. He was transferred from the Emergency Department to the Intensive Care Unit (ICU), where he was intubated and mechanically ventilated. At three weeks he was not making significant progress and was transferred to the Pediatric Intensive Care Unit (PICU) at the British Columbia’s Children’s Hospital. After two additional weeks of ventilation and intensive physiotherapy, his secretions settled, his atelectasis resolved, and he was successfully extubated to noninvasive ventilation (NIPPV). Several days later the PICU physiotherapists reported that he was losing strength in his right arm. A Computed Tomography (CT) scan revealed hydrocephalus, and on reviewing his chest X-rays, including xrays from the referring hospital, several showed that that his shunt had become disconnected high in his neck. The shunt was revised, but polysomnography continued to show hypopnea and central apnea. He was discharged on nighttime NIPPV. Prior to his respiratory illness, he had not had any of the usual symptoms of shunt dysfunction, (headache, change in level of consciousness, nausea, or vomiting), nor had he symptoms of disturbed sleep ventilation, (morning headache, daytime somnolence, or deteriorating school performance). He had had nystagmus at rest all his life, but his mother felt that this was unchanged. He had a reasonable cough and a good gag reflex and there were no fasciculations of the tongue, (which we have seen appear de novo in another patient with Chiari problems.

## Results

His upper limb strength improved gradually and his NIPPV was discontinued one year later. Successive sleep studies have been normal.

## Conclusions

Children with meningomyelocoele/ Arnold Chiari malformation, with shunt dysfunction can present with disordered sleep ventilation in the absence of either the classical symptoms of shunt dysfunction or central hypoventilation. When the clinical course of an illness in a shunt dependent child takes an unexpected turn, evaluation of shunt function is indicated.

